# C-reactive protein (CRP) recognizes uric acid crystals and recruits proteases C1 and MASP1

**DOI:** 10.1038/s41598-020-63318-8

**Published:** 2020-04-14

**Authors:** Anika Alberts, Annika Klingberg, Anne Kathrin Wessig, Christèle Combes, Torsten Witte, Korbinian Brand, Andreas Pich, Konstantin Neumann

**Affiliations:** 1 Institute of Clinical Chemistry, Hannover Medical School, 30625 Hannover, Germany; 2 CIRIMAT, Université de Toulouse, CNRS, Toulouse INP - ENSIACET, 31030 Toulouse, France; 3 Department of Immunology and Rheumatology, Hannover Medical School, 30625 Hannover, Germany; 4 Research Core Unit Proteomics & Institute of Toxicology, Hannover Medical School, 30625 Hannover, Germany

**Keywords:** Innate immunity, Crystal deposition arthropathies, Gout

## Abstract

Gout is caused by crystallization of uric acid in the form of monosodium urate (MSU) crystals, which induce a sterile inflammatory response that is hardly distinguishable from microbe-induced inflammatory responses. It is unclear, if MSU crystals (like microbes) are recognized by specific pattern recognition receptors. To identify possible soluble pattern recognition molecules for MSU crystals, we purified MSU-binding proteins from human body fluids. We identified C-reactive protein (CRP) as a major MSU-binding protein. Binding of CRP was strong enough to specifically deplete CRP from human serum. We found that CRP was required for fixation of complement components C1q, C1r, C1s and MASP1. Thus, we have identified a pattern recognition molecule for MSU crystals that links to the activation of complement. Notably, CRP does not show an even binding to the complete surface of the crystals. It rather binds to edges or distinct faces of the crystals.

## Introduction

The deposition of crystals within joints leads to inflammatory responses. The crystallization of uric acid in the form of monosodium urate (MSU) in the joints leads to gout, while deposition of calcium pyrophosphate dihydrate (CPPD) leads to pseudogout. Gout is a severe and common form of inflammatory arthritis^1^, characterized by acute attacks (flares) that spontaneously resolve. Acute gout attacks are treated with nonsteroidal anti-inflammatory drugs, colchicine or glucocorticoids. Urate lowering therapy (e.g. allopurinol) is used continuously, while the initial decrease in urate levels leads to increased risk of gout flares^2^.

MSU crystals induce inflammation by activating the complement system^3,4^, activation of myeloid cells leading to inflammatory cytokine production^5^, neutrophil activation^6^ and NETosis^7,8^, and NLRP3 inflammasome activation^9^. Thus, inflammation induced by MSU crystals is remarkably similar to inflammation induced by microbes. Gout attacks in patients may also resemble septic arthritis (fever, high CRP)^10^.

While many of the pattern recognition receptors recognizing microbes have been discovered in the last decades, it remains unclear, if specific receptors for MSU crystals exist. Receptors CD16, CD11b and especially CD14 have been shown to be involved in MSU-induced inflammatory responses^11,12^, while it is unclear if any of them specifically recognize the crystals. We have previously identified a specific immunoreceptor for MSU crystals, called Clec12A (also known as MICL and CLL-1). However, Clec12A is an inhibitory receptor that limits inflammatory responses, while potentiating type I interferon (IFN) responses^13–15^. Independent of recognition by receptors, MSU crystals activate immune cells by interacting with membrane cholesterol^16^ or inducing membrane deformation^17^. Under physiological conditions, opsonization of the crystals with complement or other opsonins may also eliminate the need for specific crystal receptors. In this study we purified MSU crystal binding proteins from human body fluids to identify potential soluble MSU recognition molecules.

## Results

### CRP binds to MSU crystals in human body fluids

**Figure 1:**
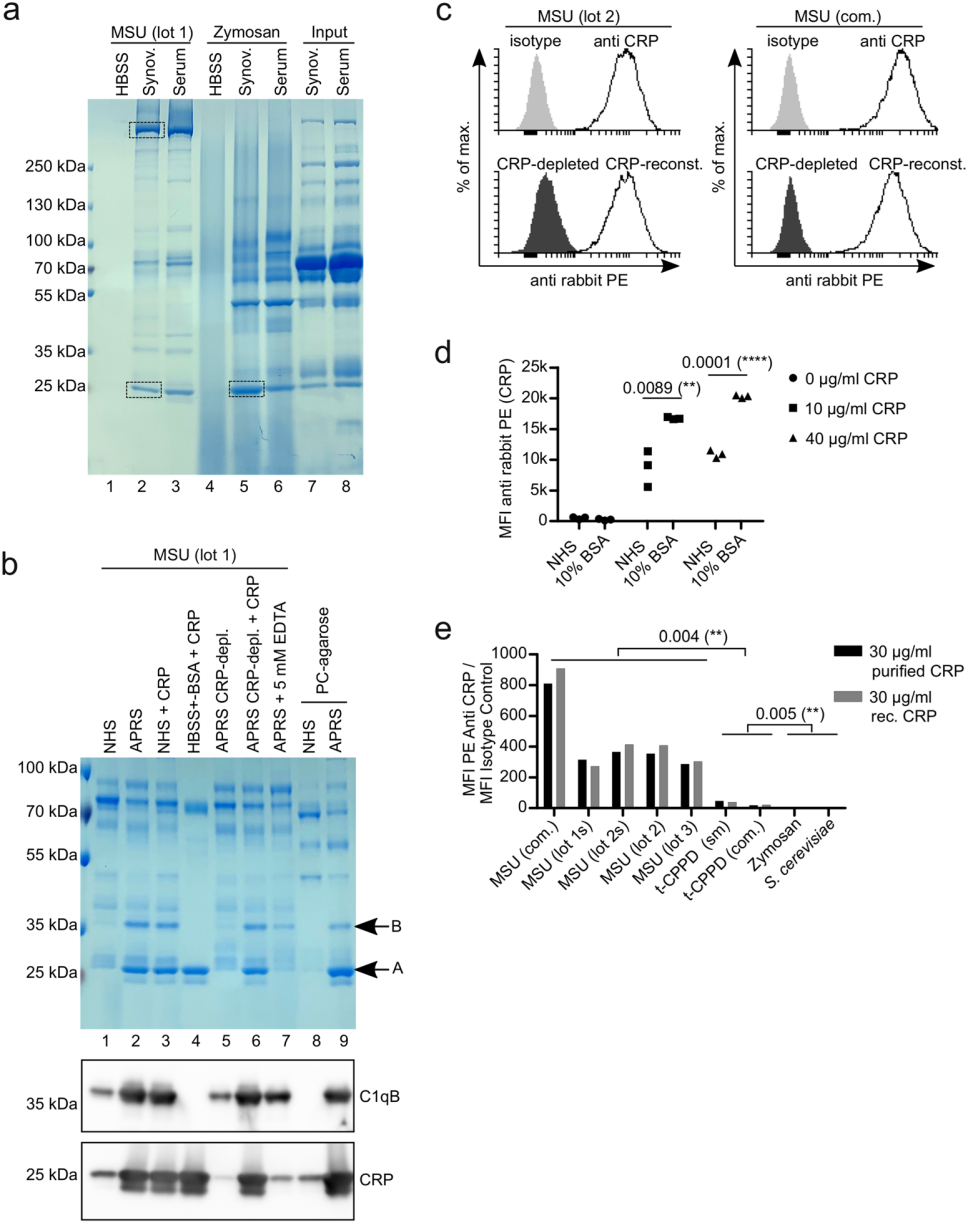
CRP binds to MSU crystals. (**a**) Synovial fluid or serum from a patient with pseudogout was incubated with MSU crystals (lot 2) or zymosan for 45 min at 37 °C. Unbound proteins were washed away and bound proteins were eluted and subjected to SDS-PAGE and visualized by coomassie. Bands excised for mass spectrometric analyses are indicated (rectangles). (**b**) Normal human serum (NHS), acute phase reaction serum (APRS; CRP around 100 µg/ml) or HBSS (always containing Ca^2+^) with or without depletion of CRP or addition of purified CRP to 100 µg/ml were incubated with MSU crystals or phosphorylcholine-agarose (PC-agarose) for 45 min at 37 °C. Bound proteins were eluted and subjected to SDS-PAGE and visualized by coomassie. In addition, the same samples were analyzed by Western blot analysis using CRP antibody (lower panel) or C1qB antibody (middle panel). **(c)** Human serum (CRP = 10.4 µg/ml) was left untreated or CRP was depleted with PC-agarose or was depleted and then reconstituted with 10 µg/ml purified CRP. All three sera were incubated with two preparations of MSU crystals (lot 1 and a commercial preparation (com.)). CRP was stained with CRP antibody and anti-rabbit-PE and analyzed using a flow cytometer. **(d)** Three different preparations of MSU crystals (lot 1, lot 2 and a commercial preparation) were incubated with either pool serum (NHS) (CRP < 0.3 µg/ml) or 10% BSA in HBSS, both with purified CRP added to the indicated concentrations. Binding of CRP to the crystals was analyzed as in **c**.  Median fluorescent intensity (MFI) is shown. **(e)** Four different preparations of MSU crystals (one commercial (com) and three self-made (untreated or sonicated (s)), two preparations of t-CPPD (commercial (com.) and self-made (sm)) and two preparations of *S. Cerevisiae* (zymosan and heat-inactivated yeast) were incubated with NHS (CRP < 0.3 µg/ml) with either 30 µg/ml purified CRP or 30 µg/ml recombinant (rec.) CRP added. Binding of CRP to the crystals and fungal particles was analyzed as in **c**. MFI of staining with CRP antibodies was divided by MFI of isotype controls. Uncropped images of gels and Western blot are shown in Fig. S3.

Since gout patients may already have formed antibodies against MSU crystals, we used synovial fluid and serum from a patient with pseudogout to purify MSU binding proteins. We additionally used the well-characterized fungal cell wall preparation zymosan as a control, as MSU crystals induce similar inflammatory responses as fungi. Both synovial fluid and serum were incubated with MSU crystals or zymosan at 37 °C for 45 min, unbound proteins were washed away and bound proteins were eluted with denaturing SDS buffer. The eluted proteins were applied to SDS-PAGE and visualized by coomassie staining (Fig. 1a). The proteins purified from synovial fluid showed a similar pattern to the proteins purified from serum. The proteins purified with MSU crystals, however, showed a mostly distinct pattern from the proteins purified with zymosan. MSU crystals purified mainly two proteins migrating at 250 kDa and 25 kDa. We excised the corresponding bands (indicated by rectangles in Fig. 1a) and identified the proteins within by liquid chromatography-mass spectrometry (LC-MS). The protein with the highest score in the band above 250 kDa was the known MSU crystal-binding protein apolipoprotein B (apoB), while in the 25 kDa band the protein with the highest score was CRP (Fig. S1a). The zymosan purification also showed a prominent band at 25 kDa. LC-MS analysis of the proteins within this band found apoA1 with the highest probability, suggesting that the 25 kDa proteins in both purifications were distinct (data not shown). We measured the concentration of CRP in both synovial fluid and serum and found around 30 µg/ml and 50 µg/ml, respectively. In line with the results from the LC-MS analysis the concentration of CRP in the body fluids was decreased after the incubation with MSU crystals, but not with zymosan (Fig. S1b), indicating CRP strongly binds to MSU crystals but not to zymosan. Since several other likely MSU-binding proteins were identified in the 25 kDa band besides CRP (apoA1, SAP, Igκ/λ; Fig. S1a), we tested if the prominent band at 25 kDa was indeed CRP. We repeated the purification of MSU-binding proteins with a set of different sera (Fig. 1b): The 25 kDa band (indicated as **A** in Fig. 1b) did not appear in the purification from low CRP normal human serum (NHS) (lane 1), but from serum from an individual with an acute phase response (APRS, CRP around 100 µg/ml) (lane 2). Addition of purified CRP to the low CRP NHS from lane 1 before purification with MSU crystals (lane 3) or to a solution of 5% bovine serum albumin (BSA) in Hank’s Balanced Salt Solution (HBSS) (lane 4) resulted in a comparable band as in lane 2. Depletion of CRP from CRP-containing acute phase reaction serum using the synthetic CRP ligand phosphorylcholine coupled to agarose (PC-agarose) selectively removed the 25 kDa band (lane 5), while reconstituting this depleted serum with purified CRP also reconstituted the 25 kDa band in the MSU purification (lane 6). In the presence of EDTA, which inhibits CRP binding to its ligands^18^, the 25 kDa band did not appear (lane 7). Lastly, when NHS or CRP-containing serum was incubated with the CRP-ligand PC-agarose, the same 25 kDa band is purified only from CRP-containing serum (lanes 8 and 9). Western blot analysis of the samples confirmed that the signal for CRP showed the same pattern as the 25 kDa band (Fig. 1b, bottom panel). Together, this proves that CRP is indeed the major constituent in the prominent 25 kDa band and thus one of the major MSU crystal-binding proteins in CRP-containing body fluids during an acute phase response.

Intriguingly, there was a second band at around 35 kDa in the coomassie-stained gel (indicated as **B** in Fig. 1b), which strongly correlated with the CRP band, but was absent when CRP was purified with MSU from a BSA solution (lane 4). Thus, it may be a post-translationally modified version of CRP or a serum protein recruited by CRP. We excised the band and LC-MS identified the protein C1qB with a higher score than CRP (data not shown). Western blot analysis using a C1qB antibody showed the same pattern as the 35 kDa band in the coomassie-stained gel (Fig. 1b, middle panel). While the Western blot analysis using the CRP antibody showed some additional bands, which may represent adducts of CRP with other proteins, none of these were in the range of the 35 kDa band (Fig. S1c). This indicates that CRP recruits C1q to the surface of MSU crystals.

To confirm that CRP binds to MSU crystals we incubated either self-made (lot 2) or commercial (com.) MSU crystals with serum containing 10.4 µg/ml CRP and stained the crystals with CRP antibody. Using a flow cytometer, we found strong binding of this antibody to both crystal preparations compared to isotype control (Fig. 1c, top panel). To test the specificity of the CRP antibody, we depleted CRP from this serum. This reduced binding of the CRP antibody nearly to isotype levels and reconstituting the depleted serum to 10 µg/ml purified CRP recovered binding of the CRP antibody (Fig. 1c, lower panel). Results from two independent MSU crystal preparations are shown in Fig. S1d.

We already showed that CRP binds to MSU crystals in a solution not containing serum proteins other than BSA (Fig. 1b). To compare the binding of CRP in the presence and absence of serum proteins, we added purified CRP to low CRP serum or a 10% BSA solution in HBSS and incubated these solutions with MSU crystals. Bound CRP was detected using a flow cytometer. Both at 10 and at 40 µg/ml, CRP showed weaker binding in serum than in BSA solution (Fig. 1d), indicating that CRP directly binds to the crystals and may even compete with other serum proteins more than it cooperates.

To test the specificity of the CRP binding, we incubated four different preparations of MSU (one was used untreated and sonicated (s)), two preparations of triclinic CPPD (t-CPPD) and two preparations of *S. cerevisiae* (zymosan and heat-inactivated yeasts) with human serum supplemented with 30 µg/ml CRP (either purified or recombinant) and analyzed CRP binding as above. As shown in Fig. 1e, both purified and recombinant CRP bound strongly to all MSU crystal preparations. CRP bound only weakly, but significantly, to both preparations of t-CPPD, but not to zymosan and *S. cerevisiae*.

### MSU crystals can be used to purify CRP

**Figure 2:**
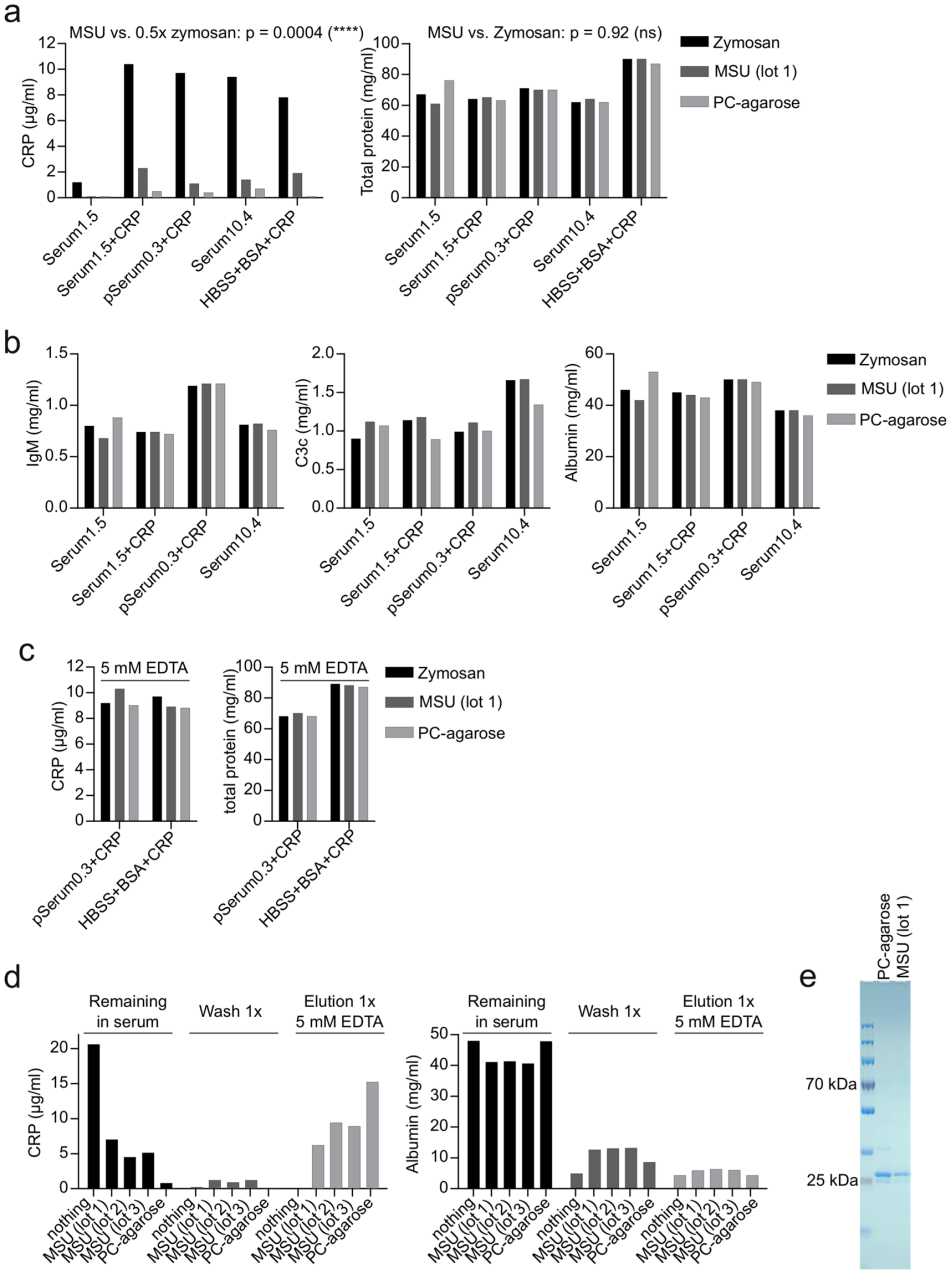
MSU crystals specifically purify CRP **(a)** 200 µl of serum of a single donor with 1.5 µg/ml CRP (Serum1.5) with or without addition of 10 µg/ml purified CRP, a pool serum with 0.3 µg/ml CRP (pSerum0.3) with 10 µg/ml purified CRP added, a single donor serum with 10.4 µg/ml CRP (Serum10.4) and HBSS 10%BSA with 10 µg/ml purified CRP added, were incubated with 3 mg zymosan, 5 mg MSU (lot 1) or 35 µl PC-agarose for 45 min at 37 °C. Samples were centrifuged and the supernatants were analyzed for CRP and total protein concentration. Using a one-sample t-test, the p-value of MSU samples compared to 50% of CRP concentration of the corresponding zymosan sample was calculated. For the difference of total protein in zymosan or MSU treated samples a paired t-test was used. **(b)** The concentration of IgM, C3c and albumin was analyzed in samples from **a** by turbidimetry. **(c)** For the indicated CRP-containing solutions the experiment was repeated in the presence of 5 mM EDTA and the supernatants were analyzed for CRP and total protein concentration. **(d)** 200 µl of pool serum containing 20 µg/ml purified CRP was incubated with nothing, three distinct preparations of MSU (5 mg each) or 35 µl PC-agarose for 45 min at 37 °C, washed 1x with HBSS for 5 min and then eluted with 5 mM EDTA in HBSS. Supernatants of each step were analyzed for CRP and albumin concentration by turbidimetry. **(e)** 100 µl of a serum containing 30 µg/ml CRP was incubated with 9 mg MSU or 35 µl PC-agarose for 45 min at 37 °C. MSU/PC-agarose was washed 5x in HBSS, and CRP was eluted by HBSS + 5 mM EDTA. Eluted proteins were applied to SDS-PAGE and proteins were visualized by coomassie staining. Uncropped image of the gel is shown in Fig. S3 (right gel). Each experiment was repeated at least once with similar results.

The immobilized CRP ligand phosphorylcholine (PC-agarose) can be used to both deplete and purify CRP from human body fluids^19^. To test, if MSU crystals may also have the ability to specifically deplete CRP from human body fluids, we incubated different human sera or a BSA solution in HBSS either with zymosan, MSU crystals, or PC-agarose. After 45 min CRP and total protein was analyzed in the supernatant. Compared to zymosan, which does not bind CRP, both MSU and PC-agarose strongly reduced the concentration of CRP but not of total protein in all solutions (Fig. 2a). MSU crystals reduced the concentration of CRP significantly more than 50%, which may be considered depletion. MSU crystals did not reduce the concentrations of known MSU-binding proteins IgM and C3, or albumin as compared with zymosan and PC-agarose (Fig. 2b). Adding EDTA to serum or the BSA solution in HBSS blocked the depletion of CRP by both MSU-crystals and PC-agarose (Fig. 2c). Together, this shows that MSU crystals are able to specifically deplete CRP from human serum in a Ca^2+^-dependent manner. This suggests that MSU may be used to purify CRP from serum. To test this, we incubated serum containing 20 µg/ml CRP with three distinct MSU preparations, washed the crystals once with Ca^2+^-containing buffer, and then eluted Ca^2+^-dependent proteins with EDTA. For each step the concentration of CRP and albumin was measured in the supernatant (Fig. 2d). CRP was strongly reduced in serum after incubation with MSU and low in the wash buffer. Nearly half of the starting concentration was recovered in the EDTA-elution. The concentration of albumin was strongly reduced in both wash buffer and in the EDTA-elution compared to input serum, indicating MSU crystals can separate CRP from other serum proteins. PC-agarose was more efficient than MSU crystals in purifying CRP, which may be due to the higher specific surface area of the porous agarose. However, when extensively washed, MSU and PC-agarose purify CRP to a similar purity (Fig. 2e). Together, this shows that the binding of CRP to MSU strongly resembles the binding of CRP to its immobilized ligand phosphorylcholine, arguing that MSU crystals could also act as a genuine ligand.

### CRP recruits C1 and MASP to the surface of MSU crystals

**Figure 3:**
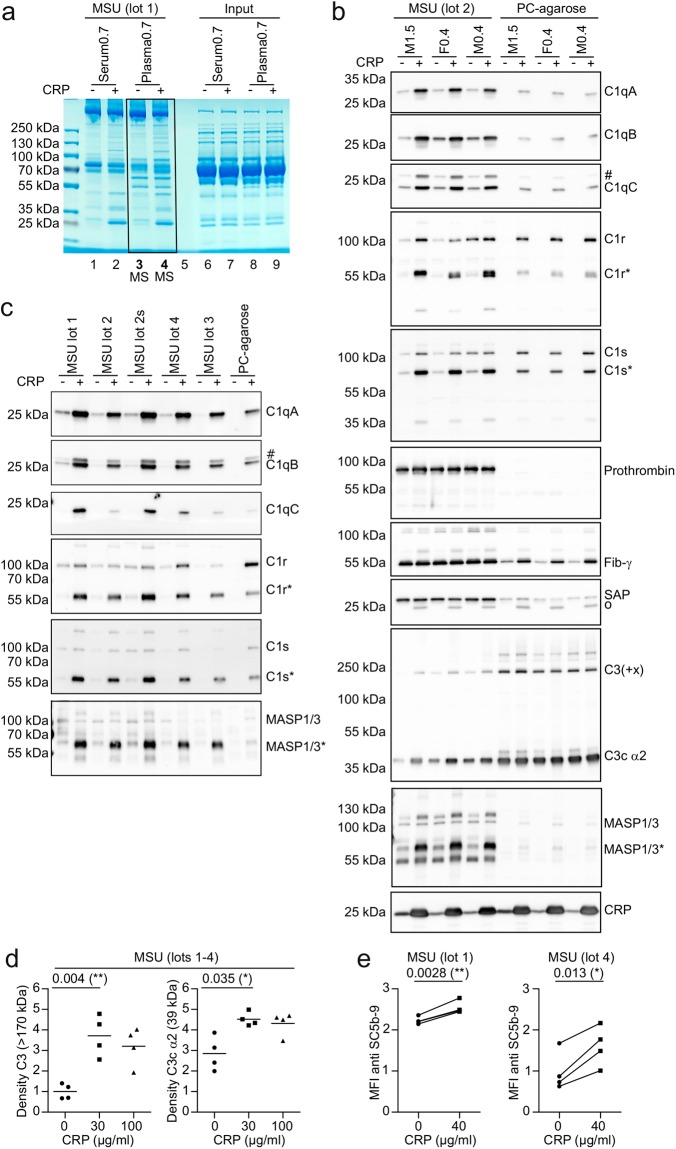
CRP recruits C1 and MASP1 to the surface of MSU crystals. **(a)** 40 µg/ml purified CRP or vehicle was added to serum and plasma (hirudin) from the same healthy male donor (CRP concentration of 0.7 µg/ml). Serum and plasma was incubated with MSU crystals (90 mg/ml) for 45 min at 37 °C. Crystals were washed extensively and bound proteins were eluted in SDS buffer, separated on a polyacrylamide gel and stained with coomassie. **(b)** 40 µg/ml purified CRP or vehicle was added to plasma of three donors, two male (M), one female (F). Numbers indicate original CRP concentration in µg/ml. Each plasma was incubated with MSU or PC-agarose and bound proteins were eluted as in **a**. Eluted proteins were subjected to Western blot analysis using the indicated antibodies. Protein names are indicated at the expected molecular weight. Cleaved/active forms of proteins are indicated with an *. Background signals due to inefficient stripping are indicated with a #. SAP antibody shows a background band at the position of CRP (o), which is likely due to cross-reactivity. **(c)** Five distinct preparations (4 lots, one of which untreated and sonicated (s)) of MSU crystals were incubated with pool serum containing vehicle or 40 µg/ml purified CRP. Bound proteins were eluted and analyzed as in **b**. **(d)** Purified CRP was added to NHS (originally containing 0.4 µg/ml CRP) to a concentration of 0, 30, or 100 µg/ml and was incubated with four distinct preparations of MSU crystals (lots 1-4) for 30 min at 37 °C. Bound proteins were eluted as in **a** and subjected to Western blot analysis using C3 antibody. Signal for full length C3 (>170 kDa) and its degradation product C3c α2 (39 kDa) were quantified by densitometry and normalized to the intensity of full length C3 in the absence of added CRP. (The corresponding Western blot is shown in Fig. S5). A paired two-tailed t-test was used to compare vehicle (0) with 30 µg/ml purified CRP. **(e)** Two distinct lots of MSU crystals were incubated in 4 individual human sera with 0 or 40 µg/ml purified CRP added for 30 min at 37 °C, extensively washed and stained with rabbit anti SC5b-9 plus anti rabbit PE. MSU crystals were analyzed using a flow cytometer. Median fluorescence intensity (MFI) of PE / 1000 is shown. A paired two-tailed t-test was used to compare vehicle (0) with 40 µg/ml purified CRP. Experiments from b-e are representative of at least 2 independent experiments. Uncropped images of the gel and Western blots are shown in Fig. S4 and S5.

In Fig. 1 we already showed that CRP enhances binding of C1qB to MSU crystals in human serum. To globally assess how CRP alters the opsonization of MSU we performed an exploratory proteomics experiment. We incubated low CRP serum and plasma of a single donor with MSU in the presence or absence of added purified CRP. Unbound proteins were washed away and bound proteins were eluted with denaturing SDS buffer and subjected to SDS-PAGE (Fig. 3a). CRP addition led to the appearance of several additional protein bands in both serum and plasma (lane 2 and 4). To identify any proteins that were enriched or depleted on the surface of the crystals in the presence of CRP, we subjected all proteins purified from plasma in the absence of CRP (lane 3) and in the presence of CRP (lane 4) to LC-MS analysis (whole lanes). Peptides were identified and quantified using MaxQuant software. A list of the 30 proteins with the highest intensity identified from lane 4 is shown in Table S1a. Of these 30 proteins, 6 proteins were increased at least 5-fold by addition of CRP (Thrombin, C1qA, C1qB, C1qC, C1r and C1s). We set out to validate these 6 proteins and 3 proteins with a high intensity that showed only a weak increase (C3, Fibrinogen, and SAP). To this end, we repeated the experiment with plasma from three distinct donors, purified both MSU crystal- and PC-agarose-binding proteins, and applied them to Western blot analysis (Fig. 3b). All five components of the C1 complex showed strongly enhanced binding to both MSU crystals and PC-agarose in the presence of CRP. For MSU crystals, binding of the catalytic subunits C1r and C1s were mainly increased in their proteolytically cleaved/active form. An enhanced binding of thrombin in the presence of CRP could not be verified, but specific binding of thrombin (in the form of prothrombin) to MSU crystals could be shown. The sample used for the LC-MS analysis experiment (Fig. 3a) did show increased thrombin binding, suggesting this discrepancy is not due to an inaccurate quantification by LC-MS but rather experimental or donor variance (data not shown).

For C3 we found an increase in C3 and a C3 degradation product (the lower band at around 39 kDa constitutes the C-terminal C3 degradation product C3c α2, according to the epitope of the used antibody) on MSU crystals in the presence of CRP, while C3 already strongly bound to PC-agarose even in the absence of CRP (Fig. 3b). In line with LC-MS results addition of CRP did not change binding of fibrinogen (γ-chain) and SAP to MSU crystals (Fig. 3b). Among the proteins identified by mass spectrometry with a low intensity, a few other proteins showed a strong increase (>10-fold) in the presence of CRP (Table S1b). From these we could verify enhanced binding of MASP1 (Fig. 3b), while MBL2 showed an inconclusive band pattern and coagulation factor VII (F7) did not show an increase (data not shown).

We repeated the experiment twice using different sera instead of plasma with similar results showing enhanced recruitment of all C1 complex subunits, MASP1 and C3 (Fig. S2; uncropped in Fig. S6, 7).

To confirm this is not due to the specific MSU crystal preparation used, we also confirmed the increased recruitment of C1 and MASP1 using five distinct preparations of MSU crystals (Fig. 3c). Note that for C1r, C1s, MASP1 and C3 we did observe protein bands that appeared to have a molecular weight greater than the full-length form of the proteins after binding to MSU crystals (e.g. Fig. 3a,b), as we saw for CRP (Fig. S1c). The reason for this is unclear, but may be due to formation of intermolecular amide bonds by C3 or transglutaminases.

Since the effect of CRP on C3 fixation was weak as compared to C1, we quantified C3 fixation after the addition of 0, 30 or 100 µg/ml CRP. We found significantly more C3 and a degradation product (C3c α2) on the surface of MSU crystals when 30 µg/ml CRP was added, while no further increase could be observed at 100 µg/ml (Fig. 3d). Our LC-MS analysis did not show enhanced fixation of complement factors downstream of C3 in the presence of CRP. However, when we tested the amount of the terminal complement complex (SC5b-9) on MSU crystals opsonized in the absence or presence of CRP, we found a small but significant increase in the presence of CRP (Fig. 3e).

Together, this shows that CRP recruits and activates C1 and MASP1 to MSU crystals, which leads to fixation of complement from C3 up to the terminal complement complex, while there seems to be additional CRP-independent mechanisms for activation and fixation of the complement factors from C3 to the terminal complement complex.

### CRP binds to distinct faces or the edges of MSU crystals

We expected the strong binding of CRP to the crystals would displace a lot of the known MSU-binding proteins. The LC-MS data suggested only a few proteins with reduced binding in the presence of CRP e.g. apoB, fibronectin and lipoprotein(a) (Table S1a). However, our attempts to verify reduced binding of apoB in the presence of CRP were inconclusive (data not shown). We also expected coating of the crystals with CRP would alter the immune cell responses to the opsonized crystals. However, when we tested inflammasome activation in a monocytic cell line (THP-1), we did not see any change in IL1β production in response to MSU crystals in the presence or absence of CRP (Fig. S2c).

**Figure 4:**
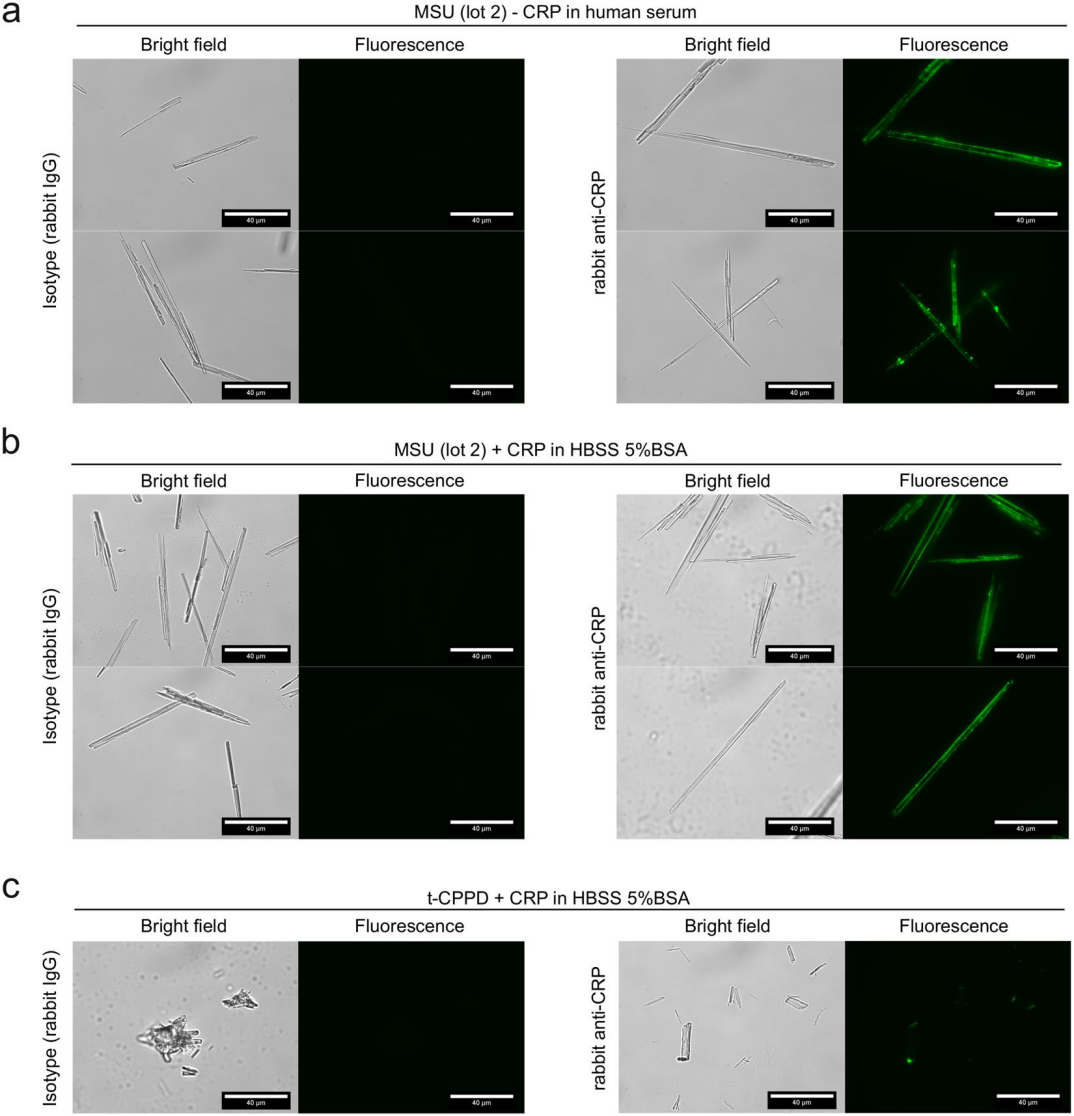
CRP predominantly recognizes the edges or specific faces of MSU crystals. **(a)** MSU crystals (lot 2) were incubated for 30 min at 37 °C with human serum (CRP 0.2 µg/ml) with 30 µg/ml recombinant CRP added. CRP binding was analyzed using CRP antibody and anti-rabbit-AlexaFluor488. AlexaFluor488-fluorescence of the crystals was detected by fluorescence microscopy; scale bar = 40 µm. **(b)** MSU crystals (lot 2) were incubated as described in **a** in 30 µg/ml CRP-containing HBSS 5% BSA with 30 µg/ml recombinant CRP added. Microscopic detection of bound CRP performed as in **a**. **(c)** t-CPPD (self-made) was incubated in HBSS 5% BSA with 30 µg/ml recombinant CRP added as described in **b**. Microscopic detection of bound CRP performed as in **a**. Each experiment is representative of at least 2 independent experiments.

**Figure 5:**
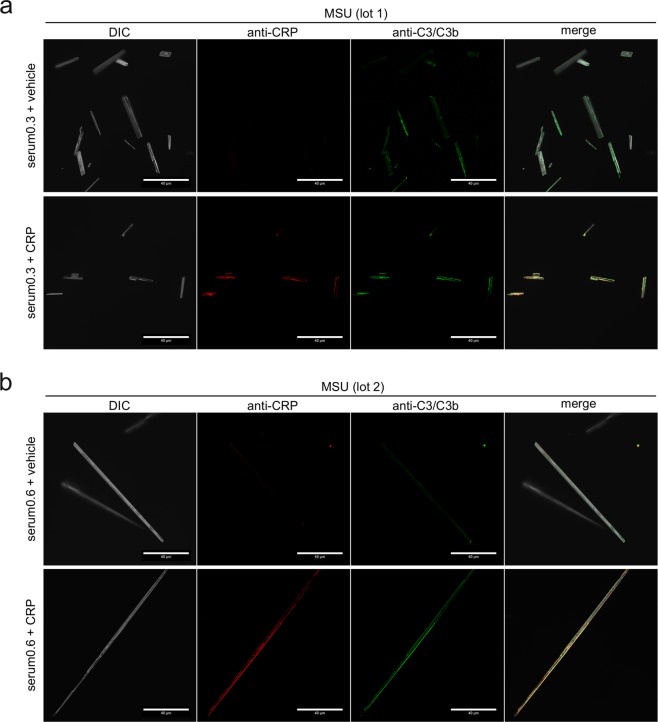
Co-localization of CRP and C3 on opsonized MSU crystals. **(a)** Confocal microscopy of MSU crystals (lot 1), which were incubated for 30 min at 37 °C with human serum (CRP 0.3 µg/ml) with or without addition of 40 µg/ml purified CRP, washed extensively and stained with rabbit anti-CRP plus anti rabbit AF568 (red) and mouse anti-C3/C3b plus anti mouse AF488 (green). DIC = digital interference contrast; scale-bar = 40 µm. **(b)** MSU crystals lot 2 were incubated in human serum (CRP 0.6 µg/ml) with or without addition of 40 µg/ml purified CRP, stained and microscopically detected as in **a**.

A likely explanation for this discrepancy would be that CRP does not bind to the complete crystal surface. We thus incubated the largest MSU crystals (lot 2) that we have generated within this project with CRP-containing serum and analyzed CRP binding by fluorescence microscopy. We indeed found that CRP was mostly located at the edges and sometimes showed a punctured localization (Fig. 4a). To test, if binding of CRP to the edges is dependent on other serum proteins, we incubated the crystals with CRP in buffer containing only BSA. Here, the binding of CRP to the edges was at least as pronounced as in serum, indicating that CRP itself preferentially binds to edges of the crystals (Fig. 4b). The areas bound by CRP may either be edges between crystal faces or narrow crystal faces. Binding to a subset of faces or the edges may explain why CRP only weakly competes with other known MSU-binding proteins. This may not be limited to MSU crystals, as a similar uneven binding of CRP was also observed for CPPD crystals (Fig. 4c). To test, if the fixation of complement induced by CRP also happens at the same sites, we co-stained CRP with C3 on the surface of MSU crystals. On two distinct crystal preparations we found co-localization of C3 at the same edges as CRP, while C3 also bound to other locations on the crystals (Fig. 5a,b). This suggests that CRP recruits C3 to the edges while an additional CRP-independent mechanism of C3 binding to MSU crystals exists.

## Discussion

In this study we were searching for MSU binding proteins that could link to the activation of the immune system. The most abundant protein on MSU crystals by a wide margin seems to be apoB, which has been described before to inhibit neutrophil activation by MSU crystals^20,21^. The second most abundant protein was CRP. It is unclear, why this has not been found before. It is likely because most studies on MSU crystal binding proteins were performed before LC-MS was available. To exclude the possibility of CRP-binding to only one potentially compromised MSU crystal preparation, we confirmed that CRP bound with similar strength to up to 6 distinct preparations, two of them from a commercial vendor. We further show binding of endogenous CRP in synovial fluid, plasma and serum from different donors as well as of CRP purified from human body fluids and recombinant CRP produced in a human cell line, indicating no special conditions or co-factors are required for binding of CRP to MSU crystals.

We show that CRP directly binds to MSU crystals with a similar binding strength and Ca^2+^-dependency as its synthetic ligand PC-agarose, which makes MSU crystals a suitable matrix for purification of CRP. We know that PC-agarose remains superior to MSU crystals for purification of CRP, because it has a higher binding capacity and because the CRP-related SAP also binds to MSU crystals. However, the fact that MSU crystals could be used for CRP purification at physiological conditions is a strong argument that MSU could be a genuine CRP ligand.

Our unbiased LC-MS experiment also showed that CRP altered opsonization of MSU crystals in a similar way as other CRP-ligands. We mainly found that CRP recruits active complement component C1 to the surface of MSU crystals. We further show recruitment of the lectin pathway protease MASP1 by CRP. This is likely due to previously described interactions of CRP with lectin pathway pattern recognition molecules like ficolin^22,23^. Together, this suggests that CRP activates both classical and lectin pathway of complement activation on the surface of MSU crystals. In line with this notion we found enhanced C3 and terminal complement complex fixation in the presence of CRP. The effect was smaller than for C1 and MASP1, suggesting that additional CRP-independent complement activating pathways are triggered by MSU-crystals or that CRP also recruits proteins that inhibit terminal complement complex formation as described before^24^. Russel *et al*. have made the observation that MSU crystals deplete complement from human serum but not from serum from about half of the patients with common variable immunodeficiency (CVID). Addition of CRP restored the ability of the crystals to deplete complement^25^, strongly supporting the notion that CRP is a pattern recognition molecule for one, albeit non-exclusive, pathway of complement activation by MSU crystals. We observed only weak binding of C1 in the absence of CRP to MSU crystals, which may be direct binding of C1 to MSU crystals^26^ or mediated by other opsonins (e.g. IgM, IgG^21^, or SAP (Fig. 3b; Table S1a)). It remains to be seen what other complement activating pathways are responsible for the CRP-independent complement activation and fixation.

CRP in its native form is a disc-shaped homopentamer. Upon binding to its ligands on damaged and apoptotic cells, it undergoes a conformational change that allows for binding and activation of C1 and may lead to dissociation to monomeric CRP^18,24,27–30^. Our finding that CRP recruits and activates C1 on the surface of MSU crystals suggests that CRP binds MSU crystals via its binding face, which leads to a conformational change that enables C1 binding to the effector face of CRP. Intriguingly, the conformational change in CRP has been postulated and shown to depend on the curvature of membranes^31,32^. We found that CRP recognizes mainly the edges or distinct faces of the triclinic crystals. The edges may provide the required curvature for binding and conformational activation of CRP. This may explain why it appears in confocal microscopy that not all CRP co-localizes with C3: Some CRP binding sites may not provide the required curvature for activation. Alternatively, distinct crystal faces may have distinct surface properties as they expose distinct sides of the urate molecule^33^. This would explain selective binding of CRP to one or more distinct crystal faces.

Similar to the preferential binding of CRP to the edges or specific sides of MSU crystals, we also found irregular binding of CRP to CPPD and it seems that the previously described binding of CRP to cholesterol crystals is also limited to certain areas of the crystals^34^.

Our mass spectrometry experiment validated most of the known MSU-binding proteins like C1, apolipoproteins, fibronectin and fibrinogen^20,21,26^. Many other proteins in the list (Table S1a) are probably also MSU-binding proteins, but we only validated two more notable novel MSU-binding proteins: Thrombin (F2) and SAP (APCS), which both bound independently of CRP. Binding of both pentraxins CRP and SAP has also previously been shown for cholesterol crystals^34^. The third member of the pentraxin family PTX3 has been shown to inhibit calcium oxalate crystallization and nephrocalcinosis^35^, and to contribute to MSU-induced inflammation in mice^36^. Thus, pentraxins seem to have evolved to detect and remove not only dead cells and tissue debris but also diverse crystallized materials.

Gout patients can exhibit very high serum concentrations of CRP during an acute attack^10^. Whether CRP binding to MSU crystals modulates the inflammation during the attack or whether this is the futile attempt of the immune system trying to remove the crystals remains to be seen. Still, the identification of CRP as a genuine MSU crystal recognition molecule more than 40 years after the conclusion that MSU crystals are the cause of gout^37^ points to a more specific interaction of the immune system with crystalline structures than previously anticipated.

## Methods

### Human body fluids

We used leftover diagnostic samples of serum and synovial fluid of a male patient with pseudogout, and a serum of a male individual with an acute phase reaction (CRP ca. 100 µg/ml). Normal human serum and plasma was obtained by drawing venous blood from 4 healthy donors (2 male and 2 female) into blood collection tubes coated with hirudin (Sarstedt, #04.1959.001) or clotting activator (Sarstedt, #02.1388). Both were collected by centrifugation at 3000 xg for 10 min after less than 30 min at 4 °C and frozen at −80 °C. All donors gave informed written consent.

Five additional single donor sera were obtained from Innovative Research (#ICERS10ML). Pool serum was generated by mixing serum of at least 3 single donor sera.

This study was approved by the Ethics Committee of the Hannover Medical School (Ref. No: 3395-2016) and was performed in accordance with the ethical standards as laid down in the 1964 Declaration of Helsinki and its later amendments.

### Adjustment of CRP concentration in body fluids

Where indicated, purified human CRP (Merck KGaA, #AG723) or recombinant human CRP (Sino Biological, #11250-HNAH) was added to serum or plasma.

CRP-depleted serum was prepared by incubating 100 µl serum with 35 µl PC-agarose (ca. 20 µl packed beads) (Thermo Fisher Scientific, #20307) with mild agitation at 4 °C over-night. PC-agarose was removed by centrifugation at 2000 xg for 2 min.

### MSU, CPPD, Zymosan and *S. cerevisiae*

Distinct crystallization protocols were used for each lot of MSU crystals: Lot 1–3: 20 mM uric acid (Merck KGaA, #U0881) was dissolved by boiling in ultrapure water containing 20 mM NaOH. After cooling to 60 °C, the pH was adjusted to a pH of 8 (lot 1), pH 9 (lot 2) or pH 7.5 (lot 3) and the solutions were sterile filtered. Solutions were kept under mild agitation at room temperature until crystals formed for 24–72 h. Crystals were harvested on a sterile filter, washed with ethanol p.a. and dried o/n at 60 °C. Crystals were analyzed by FT-IR spectroscopy and taken up in PBS at 50 mg/ml and stored at 4 °C or −20 °C. Lot 1 and 2 were used both as prepared and sonicated to reduce the size of the crystals. Lot 4: 10 mM uric acid was dissolved by boiling in ultrapure water containing 10 mM NaOH. After cooling to 60 °C, 50 mM NaCl was added and the pH was adjusted to 8.5. Crystals formed without agitation at room temperature, harvested and directly taken up in PBS. Birefringence of all preparations was analyzed by polarization microscopy. To exclude contaminations or bias of our crystals, two additional commercial lots of MSU crystals from InvivoGen were used (#tlrl-msu; lots MSU-40-02 (com.), MSU-41-01 (com. lot 2)).

Triclinic-CPPD was from InvivoGen (t-CPPD com.) (#tlrl-cppd) or was prepared (t-CPPD) as previously published by using sodium pyrophosphate as precursor instead of the potassium one^38^.

Zymosan (Sigma-Aldrich, #Z4250) was resuspended in ethanol p.a. at 30 mg/ml and stored at −20 °C. Before experiments, zymosan was washed 2x in HBSS. Dried *S. cerevisiae* were from Dr. Oetker GmbH and was rehydrated in RPMI1640 for one hour at room temperature, washed in PBS, resuspended in PBS, was heat-inactivated at 80 °C for 10 min, and stored at −20 °C.

### Quantification of CRP, albumin, IgM, complement C3, total protein, IL1β

Concentrations of CRP, albumin (HSA), IgM, C3 and total protein were determined using a cobas p701 clinical analyzer (Roche Diagnostics). Reagents used: CRP (CRPL3; #05172373 190), albumin (ALBT2; #05167043 190; note that this assay does not cross-react with BSA), IgM (IGM-2; #05220726 190), C3 (C3C-2; #05991986 190), total protein (TP2; #05171385 190). Human IL1β was quantified using Ready-Set-Go ELISA (Thermo Fisher Scientific, #88-7261-88).

### Purification of MSU-, zymosan- or PC-agarose-binding proteins

Throughout this study only HBSS containing 1.26 mM Ca^2+^, 0.9 mM Mg^2+^ and 5.5 mM D-glucose (Thermo Fisher Scientific, #14025050) was used which was saturated with sodium urate to prevent dissolution of MSU crystals.

Unless otherwise stated, 100 µl of body fluid was incubated with 8 mg MSU crystals, 5 mg zymosan, or PC-agarose (ca. 20 µl packed beads) for 30 min at 37 °C with agitation (1200 rpm). Particles were washed 6x with sodium urate-saturated HBSS by centrifugation at 2000 xg for 1 min and discarding the supernatant. Bound proteins were either eluted by adding 50 µl of 2x SDS-PAGE sample buffer (+DTT) and heating at 70 °C for 10 min or by incubating with HBSS containing 5 mM EDTA for 5 min at 37 °C.

### Analysis of CRP and SC5b-9 binding using a flow cytometer

100 µg MSU, t-CPPD crystals, or fungal particles were incubated in human serum with either purified CRP or recombinant CRP added for 30 min at 37 °C. Particles were washed in 5% BSA (Roche, #10735086001) in HBSS (as above) and bound CRP was detected by incubation with a CRP antibody (Merck KGaA, #235752, 10 µg/ml) or IgG from rabbit serum (Merck KGaA, #I5006, 10 µg/ml) as isotype control at 4 °C for 1 h, and subsequent incubation with PE donkey anti-rabbit IgG (BioLegend, #406421, 2 µg/ml) for 30 min at 4 °C. SC5b-9 was detected using rabbit anti human SC5b-9 Neo (lgG) (Complement Technology, #A227, 10 µg/ml). Particles were washed and taken up in 400 µl 5% BSA in HBSS and analyzed using a FACS Canto II (BD Biosciences) and BD FACSDiva software version 6.1.3 (https://www.bdbiosciences.com/en-eu). Flowing Software version 2.5.1 (Perttu Terho, University of Turku, Finland, http://flowingsoftware.btk.fi/) was used for visualization and analysis of data obtained.

### Fluorescence microscopy

Around 100 µg of MSU or t-CPPD crystals were incubated in human serum containing purified CRP or recombinant CRP and stained with CRP antibody or isotype control, as described for the flow cytometric analysis. C3 was stained using 10 µg/ml C3/C3b/iC3b/C3d antibody (BioLegend, clone 1H8/C3b, #846302). For microscopic evaluation anti-rabbit IgG-AF488 (Cell Signaling Technology, #4412 S, 4 µg/ml), anti-rabbit IgG-AF568 (H + L) (Thermo Fisher Scientific, #A11036, 4 µg/ml), or anti-mouse IgG-AF488 (BioLegend, #405319, 4 µg/ml) were used as secondary antibodies. After the final washing step, the particles were taken up in 100 µl 5% BSA in HBSS (as above). Images were acquired with 60-fold magnification and immersion oil, with samples mounted under cover slips on glass slides. For confocal laser scanning microscopy crystals were mounted in Immunoselect Antifading Mounting Medium (Dianova, #SCR-038447), all other samples were mounted in 5% BSA in HBSS (as above).

Images were either acquired using an Olympus IX81 inverted microscope and Cell^R^ software (version 3.2; https://www.olympus-lifescience.com/en/software/) or a Zeiss 980 Airyscan 2 in combination with Zeiss ZEN System software (blue edition version 3.0; www.zeiss.com/microscopy/int/products/microscope-software/zen.html). Brightness was adjusted, pseudo-color was inserted in the grayscale image, and scale bar was added using ImageJ (version 1.52d;https://imagej.nih.gov/ij/).

### SDS-PAGE and LC-MS

SDS-PAGE was either performed with 4–20% precast gels in tricine buffer (Merck KGaA, #PCG2008) (Fig. 1) or in Tris-based buffer system using precast gels from Thermo Fisher Scientific (#XP04205BOX) or SERVA Electrophoresis (#43289.01) according to the manufacturer’s recommendations. Note that the proteins show a slightly altered migration in the tricine buffer system (Fig. 1), especially pronounced in the slower migration of C1qB (at 35 kDa).

Gels were stained with coomassie using PageBlue Protein Staining Solution (Fermentas, #R0571). Images of coomassie-stained gels were taken with iPhone 6 s or iPhone XR (Apple) and the contrast was adjusted using ImageJ (version 1.52d).

Identification of individual protein bands was performed by Proteome Factory AG (www.proteomfactory.de).

For global identification and quantification of MSU-binding proteins in the presence or absence of CRP, eluted proteins were reduced with DTT, alkylated with acrylamide, and separated on an SDS-PAGE (4–20%, Sigma-Aldrich). Whole lanes were cut into 3 individual slices, proteins therein were in-gel digested with trypsin, and generated peptides were analyzed by an LC-MS system consisting of an Orbitrap Velos mass spectrometer coupled to an Ultimate 3000 RSLC nanoflow system (Thermo Fisher Scientific).

Raw data were analyzed with the Andromeda search engine implemented in MaxQuant software (version 1.5.3.30; www.maxquant.org)^39,40^. Proteins were identified based on a false discovery rate (FDR) of less than 0.01 on protein and peptide level.

### Western blot analysis

Proteins were separated by SDS-PAGE and transferred to nitrocellulose membranes (GE Healthcare, #10600003). Membranes were blocked in TBST + 5% BSA, incubated in primary antibody in TBST + 5% BSA o/n at 4 °C. After incubation with HRP-coupled secondary antibodies (Cell Signaling Technology, #7074, #7076 S), blots were subjected to ECL reaction. Images were acquired using ChemoStar (INTAS Science Imaging Instruments GmbH) and contrast was adjusted using ImageJ (version 1.52d). The band with the strongest signal was set to maximum (black). Quantification of Western blot signals was performed with ImageJ (version 1.52.d). Primary antibodies and dilutions were: C1qA (#11602-1-AP; 1:2000), C1qB (#16919-1-AP; 1:2000), C1qC (#66268-1-Ig; 1:4000), C1R (#17346-1-AP; 1:2000), C1S (#14554-1-AP; 1:4000), C3/C3b/C3 (#21337-1-AP; 1:1000), CFP (#17192-1-AP; 1:1000), CRP (#66250-1-Ig; 1:10000), F2 (#24295-1-AP; 1:5000), F7 (#23058-1-AP; 1:1000), FGG (#15841-1-AP; 1:2000), MBL2 (#24207-1-AP; 1:2000), SAP (#20773-1-AP; 1:2000) (all from Proteintech).

APOB (#sc-393636; 1:200) and MASP-1/3 (#sc-166815; 1:200) were from Santa Cruz Biotechnology.

### Statistical analysis

Unless otherwise stated in the figure legends, a paired two-tailed t-test was performed to compare two groups, in which either the results of a single donor serum or the results of a single MSU crystal were paired. All analysis was performed using GrapPad Prism version 5 (GraphPad Software; www.graphpad.com/scientific-software/prism/). A p value of <0.05 was considered statistically significant. *P < 0.05; **P < 0.01; ***P < 0.001; ****P < 0.0001.

## Supplementary information

Supplementary Information.
