# Supplementary material for: C-reactive protein (CRP) recognizes uric acid crystals and recruits proteases C1 and MASP1
Source: Sci Rep. 2020 Apr 14;10:6391. doi: 10.1038/s41598-020-63318-8 (PMC7156728; doi:10.1038/s41598-020-63318-8)
Supplement: Supplementary file 1 — Supplementary Information. [file 41598_2020_63318_MOESM1_ESM.pdf]

Supplementary Materials

C-reactive protein (CRP) recognizes uric acid crystals and recruits proteases C1 and MASP1

Anika Alberts[1], Annika Klingberg[1], Anne Kathrin Wessig[1], Christèle Combes[2], Torsten Witte[3], Korbinian Brand[1], Andreas Pich[4], Konstantin Neumann[1*]

[1]Institute of Clinical Chemistry, Hannover Medical School, 30625 Hannover, Germany
[2]CIRIMAT, Université de Toulouse, CNRS, Toulouse INP - ENSIACET, 31030 Toulouse, France
[3]Department of Immunology and Rheumatology, Hannover Medical School, 30625 Hannover, Germany
[4]Research Core Unit Proteomics & Institute of Toxicology, Hannover Medical School, 30625 Hannover, Germany

Supplementary Tables & Figures

Table S1: MS-identified MSU-bound proteins from Figure 3a
Figure S1. CRP binds to MSU crystals (related to Figure 1).
Figure S2. CRP recruits C1 and MASP1 to the surface of MSU crystals in serum (related to Figure 3b, c)

Uncropped images:

Figure S3. Uncropped SDS-PAGE and Western blots from Fig. 1 and Fig. 2e
Figure S4. Uncropped SDS-PAGE and Western blots from Fig. 3a, b
Figure S5. Uncropped Western blots from Fig. 3c, d
Figure S6. Uncropped Western blots form Fig. S2a
Figure S7. Uncropped Western blots form Fig. S2b

a) LC-MS-identified MSU-bound proteins from Figure 3a

Log$_2$-intensity		CRP / Vehicle	Protein name	Gene name	Known conta-minant	Verified (WB)
Vehicle	CRP					
30.5	32.0	**2.7**	Complement C3	C3		Yes
20.5	30.9	**1305.7**	C-reactive protein	CRP		Yes
29.3	30.0	**1.6**	Fibrinogen alpha chain	FGA		
27.8	29.2	**2.7**	Fibrinogen beta chain	FGB		
28.3	29.2	**1.8**	Serum albumin	ALB		
25.7	28.8	**8.7**	Keratin, type I cytoskeletal 10	KRT10	x	
28.2	28.8	**1.6**	Apolipoprotein E	APOE		
28.1	28.8	**1.5**	Serum amyloid P-component	APCS		Yes
27.7	28.6	**2.0**	Fibrinogen gamma chain	FGG		Yes
26.1	28.5	**5.2**	(Pro)thrombin	F2		unchanged
30.3	28.4	**0.3**	Apolipoprotein B-100	APOB		inconclusive
28.0	28.3	**1.3**	Coagulation factor X	F10		
24.1	28.3	**17.7**	Complement C1qB	C1QB		Yes
25.5	28.1	**6.0**	Complement C1qC	C1QC		Yes
27.4	27.9	**1.5**	Vitronectin	VTN		
29.6	27.9	**0.3**	Fibronectin	FN1		
27.1	27.8	**1.6**	Apolipoprotein A-I	APOA1		
26.9	27.7	**1.7**	Keratin, type II cytoskeletal 1	KRT1	x	
21.4	27.5	**67.9**	Complement C1qA	C1QA		Yes
27.2	27.4	**1.1**	Keratin, type I cytoskeletal 9	KRT9	x	
26.5	26.7	**1.1**	Histidine-rich glycoprotein	HRG		
24.5	26.1	**3.1**	Clusterin	CLU		
27.8	25.9	**0.3**	Apolipoprotein(a)	LPA		
24.5	25.7	**2.4**	Ig kappa chain C region	IGKC		
24.4	25.7	**2.5**	Vitamin K-dependent protein C	PROC		
21.6	25.7	**16.7**	Complement C1r	C1R		Yes
22.7	25.4	**6.7**	Complement C1s	C1S		Yes
25.0	25.4	**1.3**	Vitamin K-dependent protein S	PROS1		
25.2	25.3	**1.1**	C4b-binding protein alpha chain	C4BPA		
23.4	25.2	**3.6**	Vitamin K-dependent protein Z	PROZ		
25.8	25.2	**0.7**	Apolipoprotein A-IV	APOA4		
24.9	25.1	**1.1**	Complement C5	C5		
24.8	25.0	**1.2**	Keratin, type II cytoskeletal 1b	KRT77	x	
22.9	25.0	**4.4**	Coagulation factor IX	F9		
25.9	24.9	**0.5**	Complement C9	C9		

b) Low abundant proteins that show more than 10-fold change

20.3	23.9	**12.7**	Coagulation factor VII	F7		unchanged
18.3	21.9	**12.0**	Mannan-binding lectin serine protease 1	MASP1		Yes
nd	15.4	**nd**	Mannose-binding protein C	MBL2		inconclusive

Table S1: MS-identified MSU-bound proteins from Figure 3a
a) List of proteins with the highest intensity identified from lane 4 (in presence of CRP) using MaxQuant. Ratio of the intensity in the presence of CRP (lane 4) to the intensity in the absence of CRP (lane 3) is shown in the 3rd column. Green rows indicate proteins that are increased at least 5-fold, red rows indicate proteins that are decreased at least 3-fold in the presence of CRP. Last lane indicates the results of validation by Western blot analysis.
b) List of all remaining proteins with low intensity that show an increase of >10-fold in the presence of CRP (Proteins with a score <30 were excluded).

a

↑Family	M	DB	Accession	Score	emPAI	Mass	Cov	Match(sig)	Seq(uniq+sig)	Description
2	1	Uniprot_human	2::P02741	4915	1.78	25194	0.28	162	9	C-reactive protein OS=Homo sapiens GN=CRP PE=1 SV=1
3	1	Uniprot_human	2::P02647	1850	5.50	30759	0.61	56	18	Apolipoprotein A-I OS=Homo sapiens GN=APOA1 PE=1 SV=1
5	1	Uniprot_human	2::P04264	1036	0.55	66170	0.16	26	10	Keratin, type II cytoskeletal 1 OS=Homo sapiens GN=KRT1 PE=1 SV=6
6	1	Uniprot_human	2::P02743	858	1.75	25485	0.35	24	9	Serum amyloid P-component OS=Homo sapiens GN=APCS PE=1 SV=2
7	1	Uniprot_human	2::P02747	844	1.17	25985	0.25	24	5	Complement C1q subcomponent subunit C OS=Homo sapiens GN=C1QC PE=1 SV=3
8	1	Uniprot_human	2::P01834	800	2.22	11929	0.79	22	5	Immunoglobulin kappa constant OS=Homo sapiens GN=IGKC PE=1 SV=2
10	1	Uniprot_human	2::P02649	494	0.49	36246	0.22	7	5	Apolipoprotein E OS=Homo sapiens GN=APOE PE=1 SV=1
11	1	Uniprot_human	2::P01619	294	0.55	12663	0.22	4	2	Immunoglobulin kappa variable 3-20 OS=Homo sapiens GN=IGKV3-20 PE=1 SV=2
14	1	Uniprot_human	2::P0CF74	255	1.07	11441	0.46	7	3	Immunoglobulin lambda constant 6 OS=Homo sapiens GN=IGLC6 PE=1 SV=1
15	1	Uniprot_human	2::A0A0C4DH68	234	0.89	13185	0.21	9	2	Immunoglobulin kappa variable 2-24 OS=Homo sapiens GN=IGKV2-24 PE=3 SV=1
16	1	Uniprot_human	2::O95445	223	0.70	21582	0.16	12	4	Apolipoprotein M OS=Homo sapiens GN=APOM PE=1 SV=2

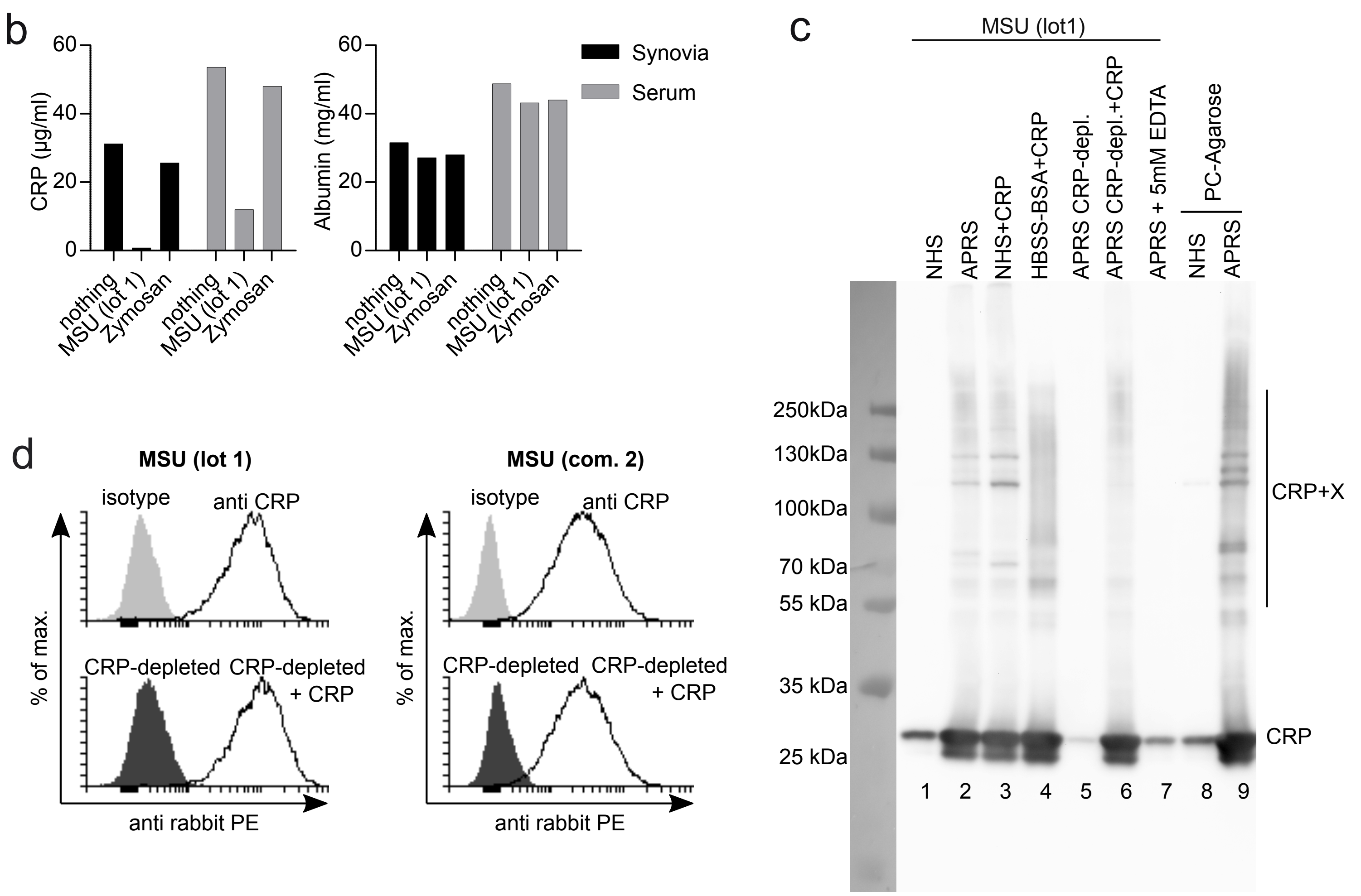

Figure S1. CRP binds to MSU crystals (related to Figure 1).

a) Proteins identified from 25 kDa band purified from synovial fluid using MSU.

b) CRP was quantified from synovial fluid and serum samples from Figure 1a before (nothing) or after incubation with MSU or zymosan.

c) Complete anti-CRP Western blot from Figure 1b.

d) MSU crystals lot 1 or commercial MSU (com. 2) were stained as in Figure 1c.

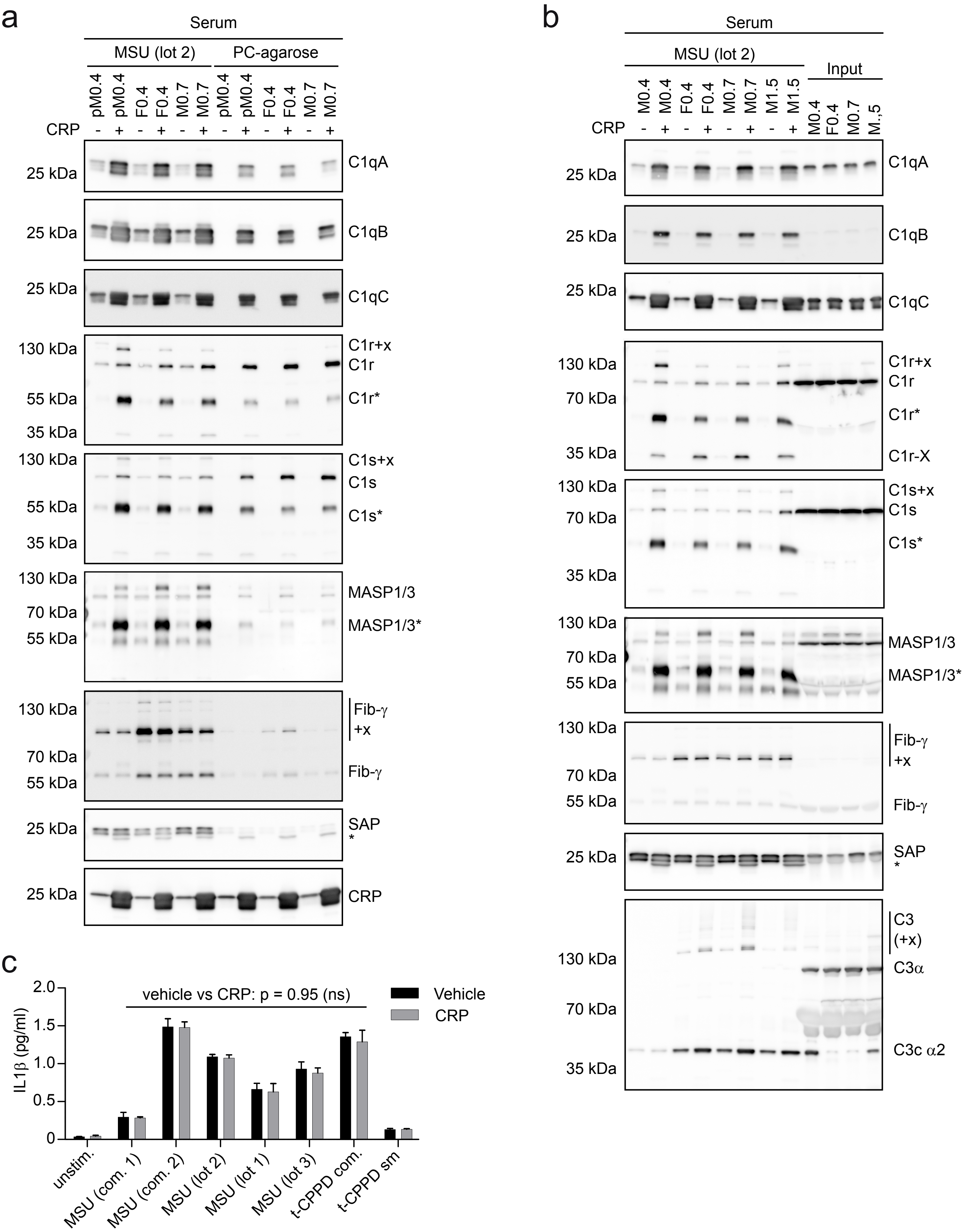

Figure S2. CRP recruits C1 and MASP1 to the surface of MSU crystals in serum (related to Figure 3bc)

a) Experiment was performed as in Figure 3b, but with serum instead of plasma.

b) Experiment was performed with four different sera as in **a**, with the input serum applied to lanes 9 – 12.

c) THP-1 cells were primed for 16 h with PMA and then stimulated for 3 h with five distinct preparations of MSU and two preparations of t-CPPD crystals that were opsonized in human serum in the presence or absence of 100 µg/ml purified CRP. IL1β was analyzed in the supernatant by ELISA. Triplicates + SD are shown. Paired students t-test was performed on all MSU crystals in the absence vs. in the presence of CRP; representative of two independent experiments

Uncropped gel from Fig. 1a

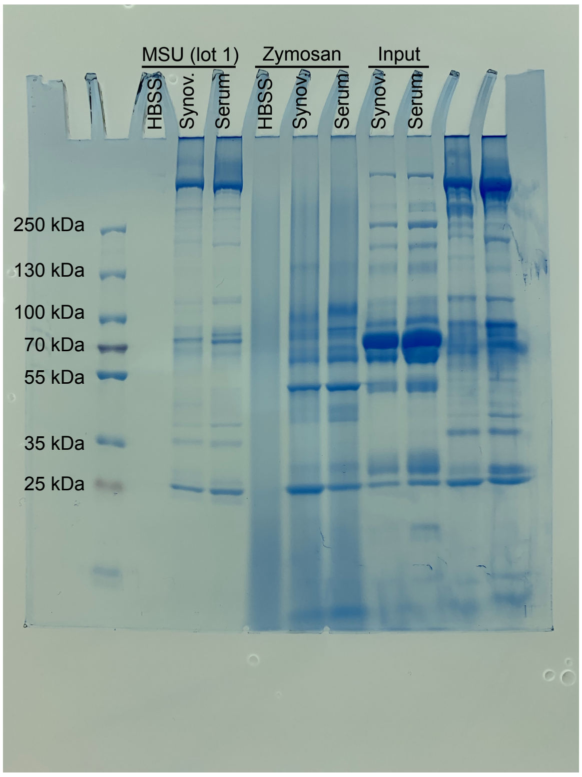

Uncropped gel from Fig. 1b and Fig. 2e

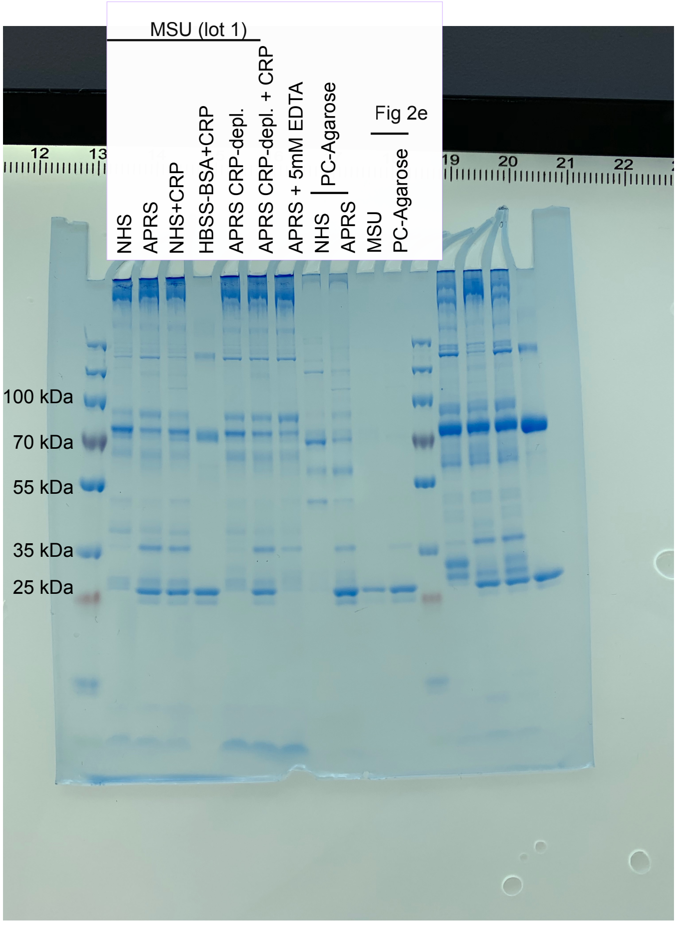

Uncropped Western blot from Fig. 1b middle panel

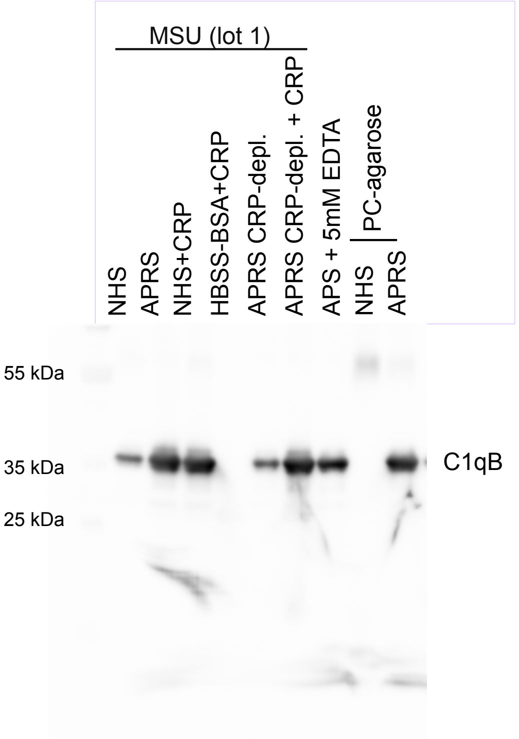

Uncropped Western Bot from Fig. 1b lower panel using anti CRP is shown in Fig. S1c

Figure S3. Uncropped gels and Western Blots from Figure 1 and 2e

Figure S4. Uncropped gels and Western Blots from Figure 3a, b

Uncropped coomassie-stained SDS-PAGE gel from Fig. 3a

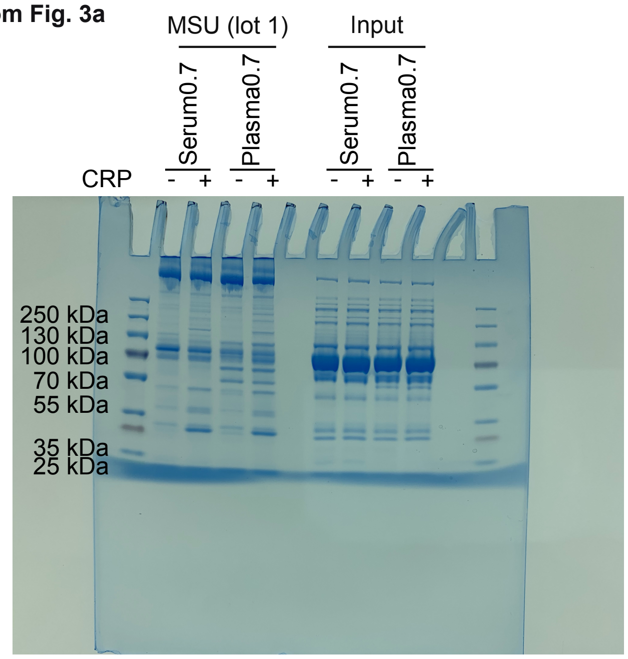

Uncropped Western blots from Fig. 3b

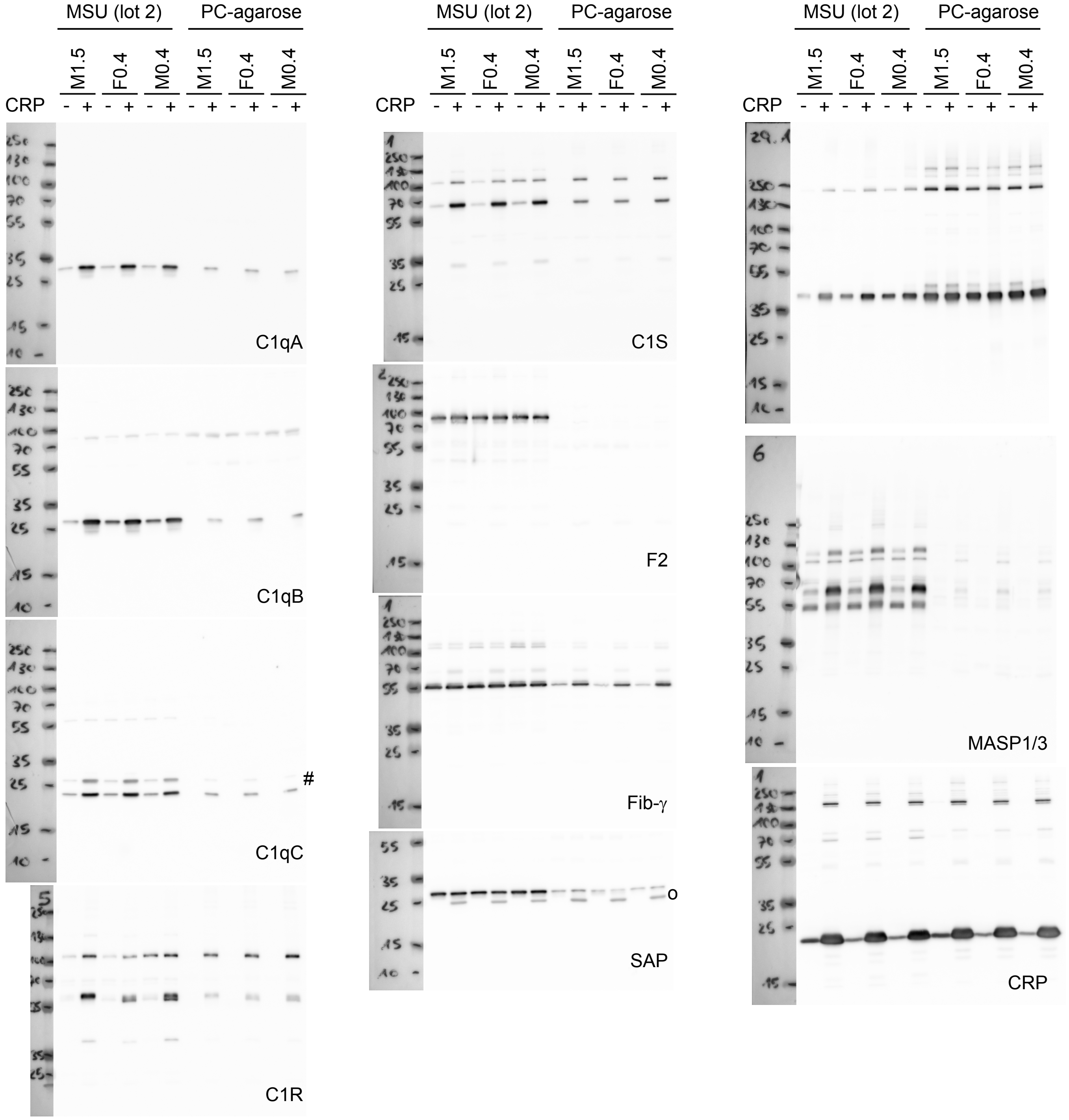

Uncropped images from Fig. 3c

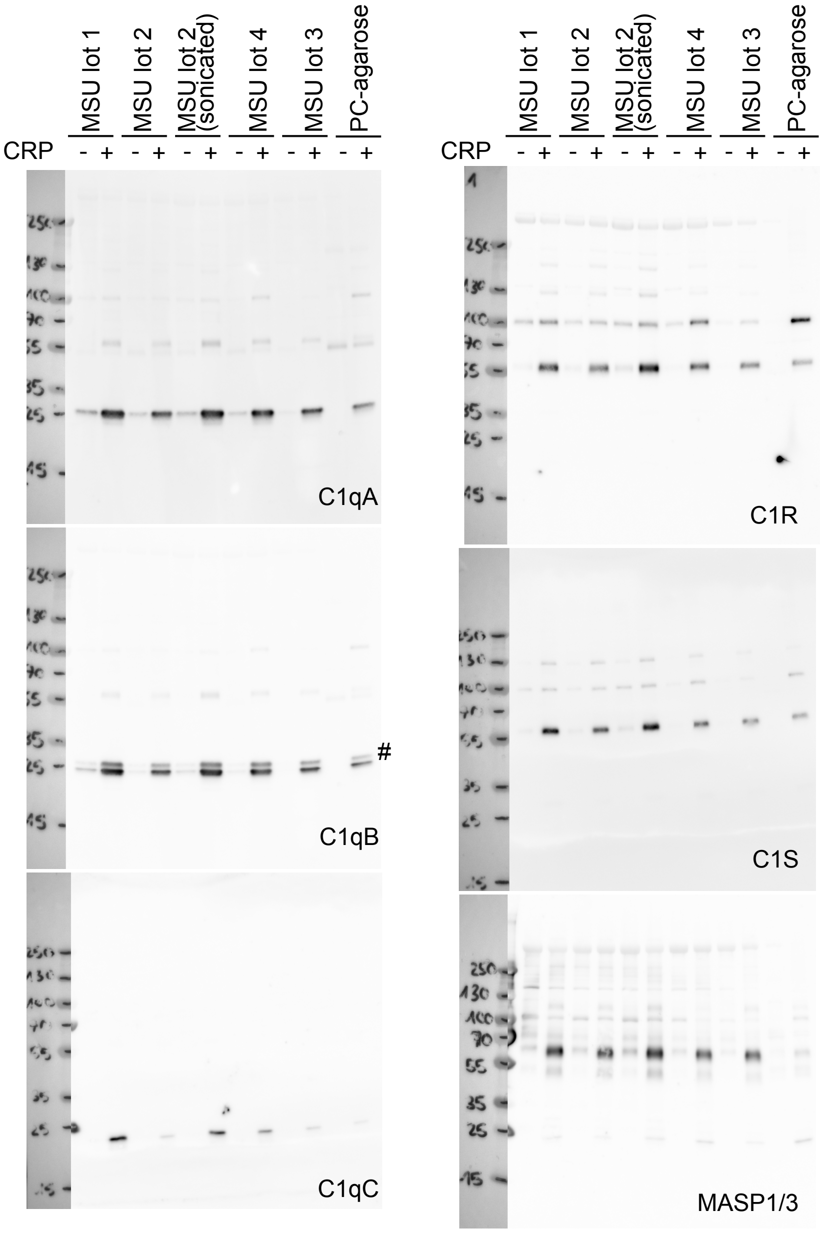

Western blot used for quantification of C3 binding in Fig. 3d

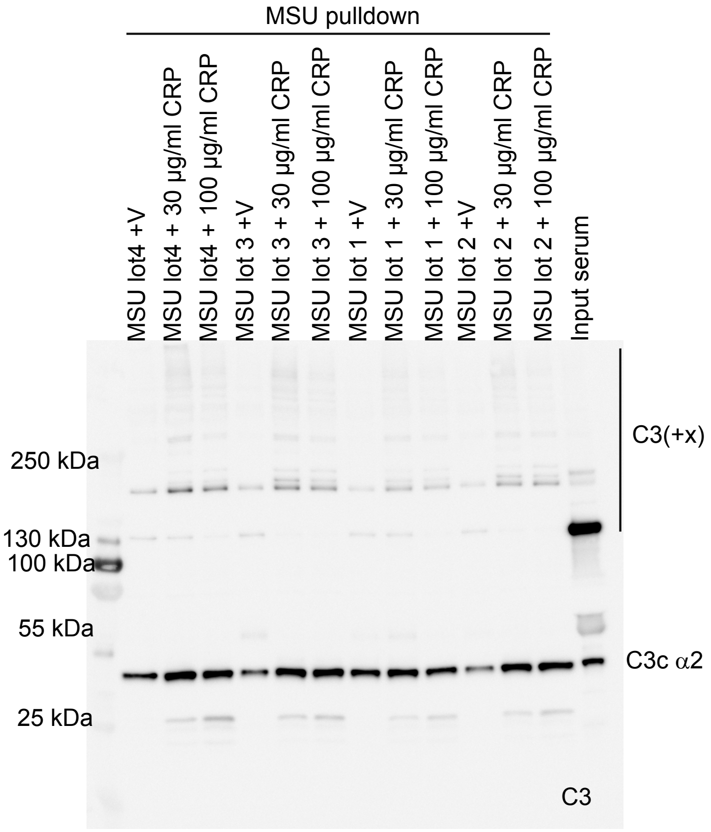

Figure S5. Uncropped gels and Western Blots from Figure 3c, d

Uncropped Western blots of Fig. S2a

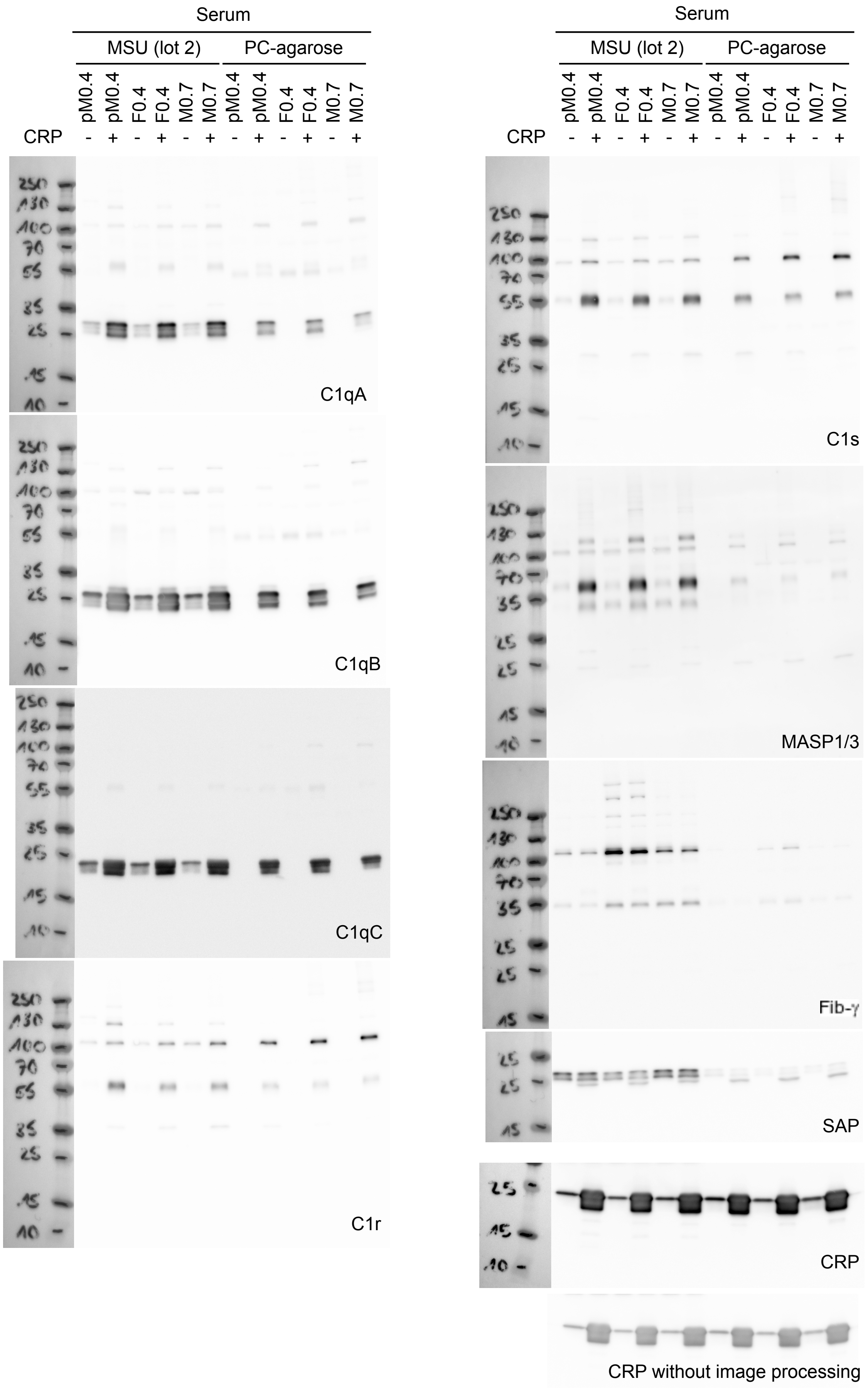

Figure S6.Uncropped gels and Western Blots from Figure S2a

Uncropped Western blots of Figure S2b

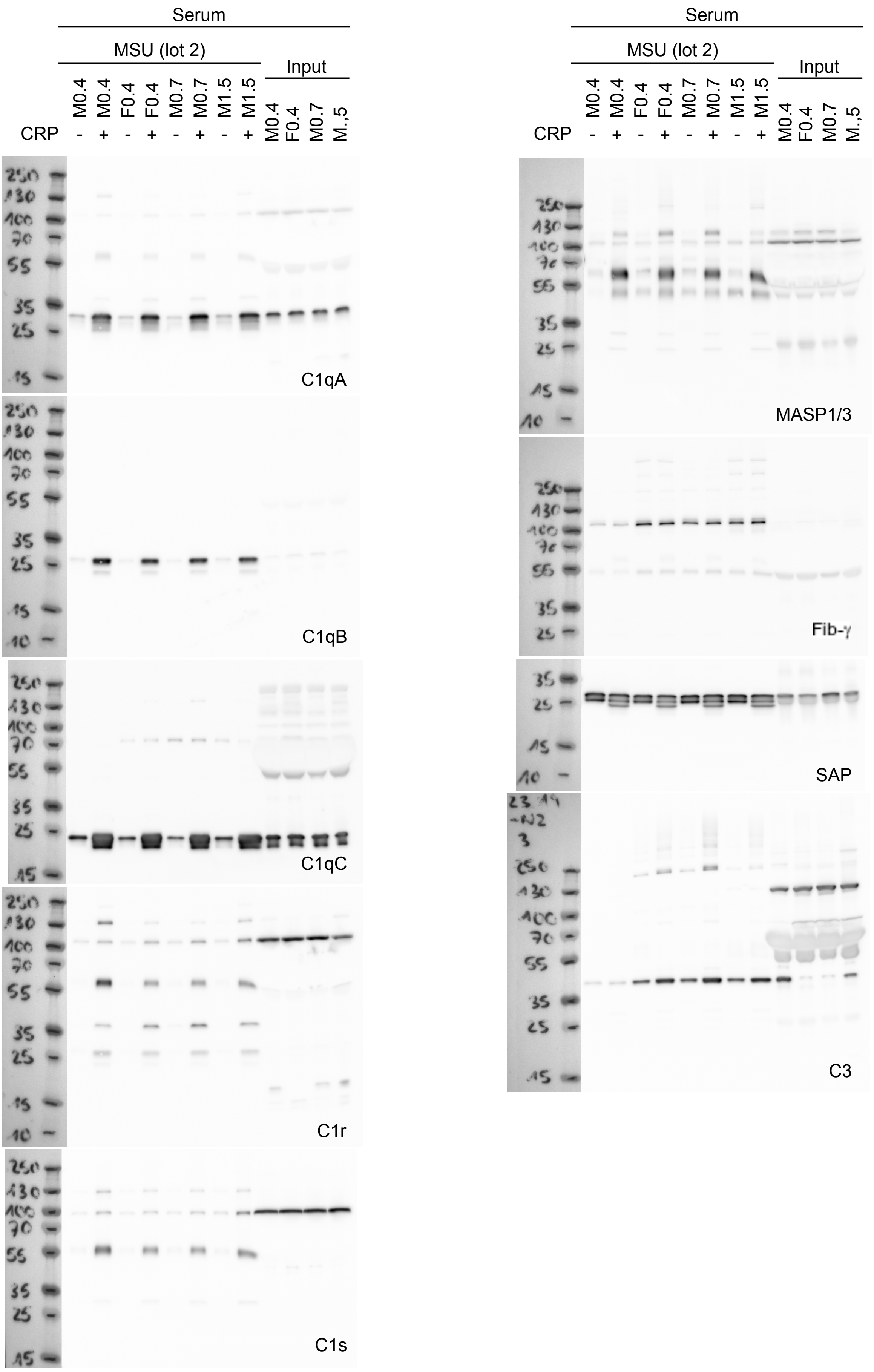

Figure S7. Uncropped gels and Western Blots from Figure S2b
